# Global Health in Swedish Nursing Curricula: Navigating the Desirable and the Necessary

**DOI:** 10.3390/ijerph18179372

**Published:** 2021-09-05

**Authors:** Monne Wihlborg, Helen Avery

**Affiliations:** 1 Department of Health Sciences, Integrative Health Research, Faculty of Medicine, Lund University, 22210 Lund, Sweden; Monne.Wihlborg@med.lu.se; 2 Centre for Environmental and Climate Science (CEC), Faculty of Natural Sciences, Lund University, 22210 Lund, Sweden; 3 Centre for Advanced Middle Eastern Studies (CMES), Faculty of Social Sciences, Lund University, 22210 Lund, Sweden; 4 Department of Languages, Faculty of Arts and Humanities, Linnaeus University, 35195 Växjö, Sweden

**Keywords:** global health, social determinants of health, health equity, environmental justice, sustainability, Agenda 2030, undergraduate nursing education, course syllabi

## Abstract

Global health challenges are likely to be aggravated in the coming years by rapid climate change and environmental degradation. To address the resulting health inequities, nurses need an integrated understanding of environmental and social determinants of health. This study adopts an explorative inductive approach to examine how global health and sustainability are expressed the course syllabi of undergraduate nursing programmes (*n* = 24) in Sweden. After excluding biomedical and other unrelated content, 67 syllabi were selected for a thematic analysis. Results indicate that global health, the social determinants of health and sustainability tend to appear in a fragmented manner in the syllabi. Global health content is often limited, relegated to elective courses, or altogether missing. A theoretical framework is lacking, and focus lies on an individual rather than structural perspective. Based on international policy, earlier studies on undergraduate nursing education and theoretical work, suggestions are made for how global health and sustainability content could be integrated into nursing education, notably by using a structural competency approach.

## 1. Introduction

Global health is a contested field of research, policy and practice, which reflects diverging conceptions of the world economic order and in the way power relationships between geographical regions and between social groups are seen [1,2,3,4,5]. Notwithstanding existing divergences in standpoints and theoretical perspectives on global health as such, in view of the urgency of global health challenges the question of how education prepares professionals to meet these challenges merits closer attention [3,6]. 

Central to international policy on global health is a concern for equity between high income countries and low-income countries. This concern permeates documents and reports emanating from the World Health Organisation [7,8], as well as international development policy as expressed, for instance, in the Millennium Goals and Agenda 2030 [9,10]. Links between global health and both social and environmental sustainability have increasingly been highlighted in recent years [4,11,12,13,14]. Besides the issue of inequities between countries, international documents such as the Ottowa Charter for Health Promotion [15]) also emphasise health equity within countries.

The overall aim of this study is to investigate how global health and global health competencies appear in Swedish undergraduate three-year nursing programmes, with a particular emphasis on social and environmental determinants of health. An explorative inductive approach was used for this qualitative study, and the framing research questions was: How is global health expressed in Swedish undergraduate nursing courses? Data consisted of all available course descriptions in programmes offered in Sweden, collected between April 2020 to August 2020, which were scanned for content relating to global health Syllabi that contained at least some global health related content were then thematised. To gain a more complete picture, the analysis included curriculum content that did not explicitly use terms such as ‘global health’ or ‘global nursing’, but which nevertheless directly or indirectly related to the objectives of international policy documents to address health equity and the social determinants of health, as well as with respect to links between health equity and the environment. To provide a basis for future curriculum development, a brief overview is additionally provided of the development of the WHO stance with respect to these interconnected issues, as well as earlier research concerning the relevance for nursing education and practice, with examples of studies on various approaches to integrating content relating to global health and health equity in nursing curricula.

In this study, we will take our point of departure in the international policy documents emanating from the WHO and other organisations with respect to health inequities, development agendas and social determinants of health [7,8,16,17], (2 considering their relationship to environmental issues, both concerning inequities between countries, and within countries. While Kickbusch’s [18] definition of global health primarily sees it as an area that focuses health issues across national borders (including action on global forces that determine health), for the purposes of this study we will follow Koplan et al. [1], who define global health as “an area for study, research, and practice that places a priority on improving health and achieving health equity for all people worldwide” ([1] p. 1995). The latter definition thus covers both more limited transnational questions, and the deeper structural issues that arise from inequities between and within countries [17,19], including within countries of the global North such as Sweden [20,21,22,23]. For the purposes of this study, we base our understanding of the term “health equity” between and within countries in Koplan et al.’s [1] definition, as comprising social and environmental determinants of health, and as being premised on both social and environmental justice, as outlined in Section 1.1 and Section 1.2 below.

### 1.1. Social Determinants of Health and Health Inequities

Health is directly impacted by social determinants of health such as income, working conditions and environmental factors [17,20,24]. It is also affected by social exclusion and disempowerment related to status, class or perceived ethnicity [25,26]. The conceptual framework [26,27], developed by the WHO Commission on Social Determinants of Health (CSDH) extends this definition to include environmental dimensions, as well as wider societal conditions that are not linked to individual life circumstances. The framework outlines the basis for health equity in international law and policy, as well as mapping the complex interrelationships between different determinants and identifying points of action. Donkin et al. [19] estimate that between 45% and 60% of the variation in health status globally can be attributed to social and environmental influences. The magnitude of impacts of social determinants of health (SDH, also abbreviated SDOH in the literature) has led to numerous calls for action and is a core concern of the UN agencies. Health is influenced by countries’ social and welfare policies [28,29], so cannot be understood as a matter of public health policy alone.

To address these challenges, empowerment of citizens and communities is crucial. Of particular relevance to nursing education is the Ottowa Charter [15], which defines health promotion not only as enabling people to increase control over their health, but to realize aspirations, satisfy needs, as well as change or cope with the environment. In WHO health strategies for Europe [21] it is stressed that people affected by policy must also be able to shape it, particularly groups that are most vulnerable.

### 1.2. The Relation between Environmental Determinants of Health and Equity

Several of these interrelated issues to which nations worldwide have committed are summarised in the Agenda 2030 sustainable development goals [9,10,30,31] and included in the definition of ‘global health’ and ‘global nursing’ proposed by Wilson et al. [3]. The strategic connections between global health and sustainability are also highlighted in Sweden [32]. 

Kruize et al. [12] connect the issue of health impacts emanating from environmental inequalities to the WHO conceptual framework of social determinants of health [27]. The material and psychosocial conditions mentioned in the SDH framework are according to Kruize et al. closely linked to aspects found in the environmental justice literature. The CSDH framework additionally addresses other aspects such as structural drivers. Environmental justice concepts can thus be enriched by the CSDH framework, but a systemic approach and more research is according to Kruize et al. [12] needed on how different determinants interact.

Environmental justice is concerned with disparities in how risks and access to amenities affect various groups (distributional justice). It also concerns ‘procedural justice’, that is, access to decision-making processes on issues that affect people’s lives and health [12]. Not only physical and psychosocial environments, but also the degree of empowerment is therefore crucial to health impacts. To tackle such issues and change policy, descriptive studies are insufficient—the causal mechanisms must also be exposed [12,14], and issues of political marginalisation and discrimination must be addressed.

Friel [14] argues that climate change will dramatically aggravate health disparities globally, and that health equity is therefore intimately connected to global economic and political systems (see Wallerstein [33,34]; Labonté and Ruckert [4]) that distribute wealth in certain regions, while disproportionally imposing adverse impacts of business-as-usual in others. Globalisation can therefore be considered a “determinant of the determinants of health” [4], while health disparities cannot be addressed without a systemic and structural approach.

### 1.3. Global Health and in Nursing Programmes

Much of the work of UN and other international bodies is directed towards national governments, and implications for nursing practice and education are therefore not always explicit. Exceptions are notably community health initiatives drawing on the Ottowa Charter [15] and Jakarta Declaration on health promotion [35], as well as rights-based approaches to health [36,37,38]. However, according to a review by Wilson et al. [3], the role nursing can play in global health goes well beyond traditional nursing skills. On the one hand, concern with societal dimensions of health involves multiple types of collaboration and awareness of complex environmental, social or economic interrelationships. Public and community health are emphasised. On the other hand, nursing takes on a dimension of advocacy through the concern with equity and access for all. The themes identified by Wilson et al. [3] thus included interdependence, collaboration, glocal thinking and action, advocacy, caring, cultural competence, respect for diversity, partnerships, equity, holistic practice and sustainability. The core nursing competencies identified by Clark et al. [39] in their review, are also part of such efforts to inform a wider discussion on implications of global health issues for the nursing profession, while Torres et al. [40,41] have developed a comprehensive list of global health competencies for undergraduate nursing programmes, intended to support curriculum development. Similarly, a list of core competencies addressing climate change has been developed by the Global Consortium on Climate and Health Education [42]. Of particular interest to teaching and learning GH, environmental justice and SDH is the concept of “structural competency” [43,44,45,46,47]. 

Several researchers have argued for the mandate of nurses in addressing health inequities globally, including through advocacy and action on policy [48,49,50]. Thus Montalvo [51] outlines clear strategies for nurses to influence policy and take part in decision-making, including building coalitions and being appointed to policy positions. As an example of an area where nurses can take the lead in community and public health nursing, a study by Ezeonwu [52] points to the role of the diaspora of African nurses. Diaspora nurses have the potential to mobilise resources, inform and improve community and public health programmes funded by countries of the global North, forming a bridge between community concerns and community and public health programmes.

Such approaches are far from generalised, however, and according to Reutter and Kushner [53] health professionals mostly continue to focus on health-care accessibility and acquired health behaviours, rather than on SDH. Reutter and Kushner argue that the role of nurses with respect to health inequities is both to provide “sensitive empowering care at the individual/community level to those experiencing inequities” and to work “to change the environmental and social conditions that are the root cause of these inequities” ([53] p. 273). They further argue that this in turn requires that nurses understand these causes, the policies that shape them and how they are experienced, in line with the concept of “emancipatory knowing” [54]. Among the major barriers to nurses’ action on SDH, Reutter and Kushner mention the “dominant ideology of individual responsibility” ([53] p. 275), and the emphasis on the “nurse-person relationship”, rather than on population health and policy intervention [35] (p. 276). Additional barriers to nurses’ action identified by Hosseinzadegan, Jasemi, and Habibzadeh [55] include insufficient professional authority, high workload and lack of resources, while their recommendations include attention to personal motivation and social engagement in recruitment.

## 2. Materials and Methods

This qualitative study has an explorative inductive research design, examining the course syllabi in 24 of the 25 Swedish universities, university colleges and independent university colleges where undergraduate nurse education is offered. One private institution does not have publicly available course syllabi and declined to share their syllabi for the study.

It should be noted that this is not a curriculum evaluation per se but aims to contribute to understanding of educational content in undergraduate nursing curricula and their orientation concerning the intentional meaning and understanding about global health. The documents included in the analysis are all official institutional documents. Course syllabi are publicly available online on institutions’ websites, as are faculty and institutional regulations and additional course information. Besides the course syllabi, numerous other curriculum documents were consulted as a background, including course goals and forms of examinations related to nursing undergraduate programmes. The literature in the reading lists was also examined, particularly the introductory textbooks to nursing, and works on public health, health promotion, health equity or ethics.

Inductive explorative analysis of data was applied [56,57,58,59,60]. A thematic approach [60,61,62] was used to organise the data and show patterns based on the intentions expressed in the wording of the course syllabi and to interpret the meanings. In line with Bowen [60], the analysis of the data started by skimming the material, getting an overall picture, and excluding material that was not related to the research questions in focus. The material was read through several times before relevant material was interpreted. As Bowen points out, the process has similarity with both content and thematic analysis, but differs from concept analysis.

### 2.1. Study Setting

Nursing is a regulated profession in Sweden, governed by the National Board of Health and Welfare. Education is at tertiary level, comprising three years of study with a total of 180 Hp (higher education credits), and overarching learning objectives are stipulated in the Higher Education Ordinance 1993:100 [63]. HEIs are obliged to provide students with programme descriptions and course syllabi that specify course and programme content, learning aims, as well as the forms of examination. The capacity of HEIs to deliver adequate education and grant qualifications is regularly evaluated by UKÄ, the Swedish Higher Education Authority. HEIs that deliver nursing education are however largely free to decide on curricular content and the structure of their programmes. All undergraduate nursing programmes comprise clinical practice periods distributed across the three years of study.

### 2.2. Data Collection

The data were downloaded from all available nursing programmes. The course syllabi related to each programme were collected during the period April to August 2020. Some of the updated syllabi were not available online at the time of our data collection. We made personal contact with teachers responsible for these courses and were provided with the latest syllabus available. In several cases a HEI simultaneously offered the first semesters of a revised programme and courses from an earlier programme. In such cases, our guiding principle was to always use the latest syllabus version available. To maintain anonymity, the 24 higher education institutions in Sweden offering undergraduate nursing programmes included in this study are numbered in random order.

All 369 course syllabi of the undergraduate nursing programmes were read in full text, together with their required reading lists the 67 course syllabi that mentioned anything pertaining to global health (GH), social determinants of health (SDH) or environmental/sustainability issues were included in the study for further analysis. These syllabi ranged from 1.5 to 30 Hp (Higher education credits, 1 Hp equal 40 hours’ study). For the purposes of this study, we have used the definition of global health by Koplan et al. [1] and operationalised the term “health equity” as comprising social and environmental determinants of health, based on social and environmental justice. The analysis involved several stages, starting with immersing ourselves by repeated reading and searching for meanings related to GH and identifying meaning units rather than codes.

### 2.3. Stages in the Process

The analysis was carried out in different steps:

- Scanning the 369 course syllabi and their associated reading lists, using key words to identify any content directly or indirectly related to GH;
- Dividing syllabi into courses dedicated to GH concerns (according to course names), and courses dedicated to other topics and where GH content was incidental (presented under Traces in the results);
- Assessing how many core elements of GH were included in each syllabus;
- Analysis with respect to two aspects—focus on the individual and theoretical content—that had not been considered in the initial choice of key words, but which appeared in the closer reading of the syllabi;
- Thematic grouping of content related to sustainability and environmental concerns.

#### 2.3.1. Sorting the Data

This involved exploring data and achieving familiarity through skimming and guided by an explorative approach, including our main focused meaning(s). As a first stage, clearly non-relevant data were excluded. Courses with any wording about ethics, equality, rights, sustainability, environment, international aspects, public health, health promotion, society, gender, culture or determinants of health were selected for further analysis (*n* = 67), while only purely biomedical/technical courses with no such wording, or no mention or wording about GH, were excluded (*n* = 302). Two syllabi for placement periods abroad were included in the analysis, although they did not otherwise have wording related to GH. One of the 24 programmes did not have any syllabus with GH related wording, and no syllabi are included from this programme.

This reading was conducted individually by the two authors as well as in collaboration. We then approached the material to identify descriptions concerning GH shown in the various course syllabi, to at a later stage identify specifics concerned with the intentional meaning given to GH in all undergraduate nursing programmes. All selected course syllabi were explored, identifying the subject of GH, thus describing the interpretation, meaning(s) and understanding(s) as represented in the various curricula. This process made it possible to identify several meaning units that formed clusters at a later stage.

#### 2.3.2. Analysing the Data

While content clusters were derived inductively from the course syllabi, WHO and academic definitions of GH (see Section 1 above) were then used to establish what we call ‘core elements’ of GH: (i) recognising the significance of social determinants of health, (ii) addressing health inequity in practice, (iii) understanding health as a societal and public health concern, (iv) considering health disparities both globally and within countries, and (v) including environmental or sustainability aspects. These core elements were then used to distinguish between fragmented mentions in the syllabi of aspects that could be related to GH, and an actual orientation to GH as defined in the literature.

In deepening the analysis, the course syllabi also revealed two perspectives that appeared to be common: relating differences in health to norms and/or intersectional perspectives that focus on: (vi) nurses’ interaction with individual patients (e.g., through a person/individual-centred approach), and (vii) revealing that theory on global health appeared to be missing. We therefore added these additional two perspectives to our analysis, as well as examining in further detail, (viii) how environmental and sustainability issues were expressed in relation to GH.

The search for meaning units and marking of meanings (words/concepts/sentences), individually and by both authors making notes, involved continuously comparing differences and similarities in our interpreted meanings throughout the analysis process. Based on the data’s characteristics, various themes emerged.

#### 2.3.3. Organising the Data

Organising data in clusters of meanings created a preliminary outcome showing different themes and involvements. Organising and validating the data involved scrutinising clusters of meaning units individually and by both authors collaboratively, identifying themes that describe whole meanings of GH. The outcome can be described in four general themes and became subject to refinement by judging the themes’ internal consistency. The main themes are ‘Public Health’, ‘International Health’, ‘Health Promotion’ and ‘Health and Caring’ (*n* = 26), described below in the results (Section 3.1, Section 3.2, Section 3.3 and Section 3.4).

Course syllabi that did not clearly focus on GH were then selected for analysis in a second stage. These syllabi (*n* = 41) contained one or several sentences/phrases mentioning the analysed aspects and are grouped in Section 3.5 ‘Traces’ below.

A separate Section 3.6 in the results labelled ‘Additional focus’ is reserved for describing how theory, the relation between societal and individual perspectives, as well as how sustainability and environmental issues were expressed in all the 67 analysed course syllabi in relation to GH.

## 3. Results

With few exceptions, GH appears to be overall a marginal concern in the undergraduate nursing programmes that were investigated. By contrast, aspects of GH concerns could be found in some of the specialised nursing courses at master’s level, and a few of the HEIs had a range of global health programmes at master’s level, open to different health professions. Programmes and stand-alone courses beyond undergraduate level were not included in our study.

Of the 67 undergraduate course syllabi examined, 26 were dedicated to global health themes, while 41 contained at least some elements or wording of relevance to global health. In all 302 course syllabi could not be related to global health in any way and were therefore excluded from the analysis. Overall, the reading lists for the analysed syllabi contained very few works relevant to GH except the elective courses with a global or international health focus, but even here, materials were extremely limited.

Each nursing programme has been assigned a number, according to the first order of appearance of a course syllabus from its programme in Table 1 below. The course syllabi have been listed according to study year in the programme and number of core elements included in each syllabus. If more than one syllabus concerned GH, the number of course syllabi for that year is indicated in brackets.

Of the examined programmes, only two had any syllabus containing all core elements of GH. Both of these were dedicated to Public Health; one of these was oriented towards person-centred care, while the other stressed culture as a cause for health inequity, rather than social determinants of health. The majority of the syllabi thematised under the headings Public Health, International Health, Health Promotion and Health and Caring (Section 3.1, Section 3.2, Section 3.3 and Section 3.4), included three to four of the GH core elements. Syllabi listed under the heading Traces (Section 3.5) dealt with GH concerns in a more fragmented manner, bud more than half included at least one of the core elements.

In the following thematic descriptions, illustrative excerpts of wording in the syllabi are provided. These were translated from Swedish by the authors. Tables in Section 3.1, Section 3.2, Section 3.3, Section 3.4, Section 3.5 and Section 3.6 do not indicate number of course syllabi, but only which programmes are concerned.

### 3.1. Public Health

In all, five of the programmes had courses (six syllabi) on public health. These courses were all mandatory. One course syllabus mentioned theory of health promoting behavioural change, and one course mentioned concepts, theories, and models, in public health and epidemiology. The remaining courses did not mention theory. Two of the programmes were based on a person-centred approach, one mentioned health promotion in relation to individual health, while one mentioned health promoting behavioural change. Only one programme (2) emphasised societal and structural factors.

Concerning the core elements of global health, only two of the programmes (1, 2) covered all key elements (see Table 2 below). However, one of these was based on a person-centred approach, while the other only mentioned “traditions, culture, norms and health literacy” as causes of health disparity. None of the courses explicitly mentioned social and health policy, although “structural conditions” and income were alluded to (“socioeconomic groups”). Only one syllabus mentioned the effect of the environment and lifestyle on health, one mentioned Agenda 2030 and one mentioned sustainability.

### 3.2. International Health

Considerable variation was found across the programmes concerning how the global dimension was expressed in the course syllabi. In all, seven of the programmes had courses (nine courses) on international health (e.g., global nursing, international nursing). Of these, six programmes only had elective courses, generally involving a study period abroad, while one programme had two mandatory courses on international health.

Two syllabi took their point of departure in an individual approach (person-centred respectively individual approach and family perspective). Only three of the syllabi included any mention of theory. All these mentioned “concepts” in global health. One gave no further specification, one spoke of theories of care, medicine and public health work, while the third was a stand-alone course and referred to course literature (a textbook concerning the right to health). The theoretical underpinnings of the content of the remaining syllabi were thus implicit.

None of the programmes covered all core elements, and two syllabi with elective study periods abroad did not contain any core elements (see Table 3). Social determinants of health were explicitly mentioned in one syllabus (11) and were indirectly referred to in two others (2, 10). Environmental determinants of health were not mentioned, but four of the syllabi referred to Agenda 2030. The public health content ranged from knowledge concerning typical diseases globally, to knowledge on health systems in host countries.

### 3.3. Health Promotion

In all, six of the programmes had courses (six syllabi) on health promotion. These courses were all mandatory. Societal aspects were only mentioned explicitly in one of the courses but were referred to indirectly in the other four courses by mentioning public health (13) and/or by mentioning aspects relating to determinants of health (5, 12, 13, 14). All five courses focused on health promotion in the interaction between nurses and individual patients, though only two of the courses explicitly mentioned the ‘individual level’ in the wording (8, 12). Theory was mentioned in two of the courses, concerned health promotion, and involved life-style factors and nurses’ guidance for patients’ self-care in individual conversations, respectively. These theories thus emphasise individual responsibility for health, rather than considering social determinants.

Concerning the core elements of global health, none of the syllabi covered all elements (see Table 4). One course (13) mentioned a global dimension, one syllabus (8) explicitly mentioned equality, while one syllabus (12) included a textbook on inequality. Four of the syllabi (5, 12, 13, 14) mentioned sustainable development, and one explicitly included health impacts of the physical and psychosocial environment (8).

Besides the courses that focused on international health, public health or health promotion (see Section 3.1, Section 3.2 and Section 3.3 above), content relating to GH could be found in various other course syllabi. In those cases that a significant amount of the course content related to GH issues, the content is summarised here in the table Health and caring, whereas in those cases where only some occasional wording was found, the content has been summarised in Table 6 under the heading Traces (Section 3.5).

### 3.4. Health and Caring

Significant GH related course content was found in five course syllabi. Of these, four were mandatory, while the fifth was elective and involved a study-abroad period.

Concerning theory, one of the courses had recent textbooks on global health and sociology for nurses, and one had a textbook on international health from 1989. Two of the courses stressed an individual approach. The third course adopted a more structurally oriented approach, but at the same time framed international health as a cultural matter and had a textbook on culture and health.

None of the courses described in this section covered all core elements (see Table 5). Only two of the syllabi mentioned equity/inequity. Two of the syllabi made no mention of environment or sustainability, one mentioned sustainable development, two mentioned Agenda 2030, one spoke of impacts of climate change, while one spoke of the environment and lifestyle.

### 3.5. Traces

Course syllabi (*n* = 41) grouped here under the heading Traces (Table 6) contain at least some wording relating directly or indirectly to GH (see Section 2.3.1 above). Although these course syllabi did not contain substantial GH content, several (*n* = 23), distributed across 11 of the programmes, included core elements.

### 3.6. Additional Focus of Analysis

Besides the thematic analysis above, three additional areas were focused. Two of these—(v) individual approach and (vi) theory on global health—were identified inductively after data perusal, while the third—(vii) environment/sustainability content—is motivated by the emerging literature calling for integration of environmental and social determinants of health, on the one hand, and by the implications for nursing education of recent international agreements, in particular Agenda 2030 [9]. 

#### 3.6.1. Societal Versus Individual Perspectives on Global Health and Health Equity

Based on the wording found in the course syllabi, we discerned two main perspectives in the way global health and health equity were expressed (Table 7). On the one hand, both determinants of health and approaches for addressing ill health could be expressed as societal concerns. On the other, they could be approached from an individual perspective, placing responsibility on patients and individual nurses. Some programmes tended towards primarily one or the other of these dimensions, while others encompassed both. An individual, person-centred approach was apparent in 16 of the investigated programmes. By contrast, although several of the programmes included a dedicate course on public health, structural issues remained marginal, and a theoretical framing for global health was virtually absent in the wording.

#### 3.6.2. Framing of Sustainability and Environmental Issues in Relation to Global Health

The UN SDGs are in Swedish referred to as the ‘global goals’. They have been included in our analysis, as an expression of global consensus on a prioritisation of equal health. In the course syllabi, focus can lie on social or environmental dimensions, their interrelationships, but also be limited to SDG 3 (Good health and well-being), with little or no attention to systemic aspects of GH or concerning the social and environmental determinants of health. Of the 24 programmes analysed in this study, 16 referred to sustainability or the environment in some way, while such content was altogether lacking in 8 programmes. Of those that did have some wording, 13 mentioned sustainability or sustainable development, only four used the wording ‘environment” (with reference to physical or psychosocial environment), while seven explicitly referred to Agenda 2030 or the SDGs. None of the programmes had dedicated mandatory courses at undergraduate level concerning environmental and sustainability issues in nursing and health care systems, and such courses were not listed as electives. However, we have not investigated whether students had the option to choose other electives beyond those that were listed.

The comprehensive overview of wording in the analysed course syllabi concerning sustainability, sustainable development, Agenda 2030 and the environment (Table 8) shows that several syllabi relate sustainability to global concerns and/or health inequities (Table 9). A few syllabi mention environmental impacts on health, but overall emphasis is rather on social and economic aspects. Impacts of health systems on the environment are in some of the syllabi considered with respect to safe management of pharmaceuticals and antimicrobial resistance. Just as for the SDH, however, a more structural understanding appears to be lacking, and none of the syllabi discuss, for instance, preventive approaches or more labour-intensive care as strategies for reducing use of pharmaceuticals.

## 4. Discussion

In this study, we examined all course syllabi (*n* = 369) of the 24 Swedish HEIs that offer undergraduate nursing programmes. Of these, 67 course syllabi contained some wording that related directly or indirectly to global health and were therefore selected for further analysis. Only 26 were dedicated to global health themes (here labelled Public Health, International Health, Health Promotion and Health and Caring), while 41 syllabi had another focus, but nevertheless contained some global health related content (here labelled Traces). If only mandatory courses had been included in the analysis, the number of dedicated courses would have been 18. Seven of the eight elective courses had headings related to International Health, and most included the option of a study-abroad period.

To assess to which extent courses contained content that could be related to the main issues underlined in WHO documents and the literature, we grouped the 26 course syllabi of the themes according to the number of core elements of GH (see Section 2.3.2) that they contained. Only two contained all core elements, four did not contain any core elements, while the remaining 20 syllabi contained 1–4 core elements. The analysis thus suggests fragmentation in two respects. Firstly, merely including isolated aspects of content in courses that were not dedicated to GH concerns, without supporting literature or an overarching framework, is unlikely to offer students opportunities to understand systemic interconnections and the structural forces that drive health inequity. Secondly, even courses dedicated to GH concerns rarely included all core elements. Therefore, these courses also afforded limited opportunities for an integrated systemic understanding GH or learning how to translate it into practice. The fragmentation was exacerbated by lack of a theoretical framework. A more coherent vision for the curriculum is therefore called for (see Valderama-Wallace and Apesoa-Varano [64])

In the Swedish undergraduate nursing programmes that we analysed, aims in the programme descriptions tend to include the aspects in national nursing qualifications requirements [63] with direct or indirect relevance to global health. These include “knowledge of social circumstances that affect the health of children, women and men”; to “promote health and prevent ill-health”; and “a holistic approach to individuals informed by the relevant disciplinary, social and ethical aspects and taking particular account of human rights”. However, course syllabi suggest that not only GH, but social and preventive medicine remain deprioritised. Health issues of high-income countries are dominant in course descriptions, and international communication is limited to English. Issues concerning vulnerable populations in Sweden were in the course syllabi almost always addressed as individual concerns. More generally, societal issues were strongly backgrounded in the course descriptions. Dominant descriptions of health and health care expressed through the intentions of the course syllabi instead emphasised individual responsibility and lifestyle. The impact of environment was expressed as immediate care settings, rather than environmental impacts at societal level or through work and living conditions. Most course syllabi comprised some intentions referring to ethics, but in ethics content and reading lists, little focus was placed on questions of social and environmental justice.

### 4.1. Individual Perspective

The results of our study showed great emphasis on “person-centred” individualised care in many of the course syllabi, with a focus on nurse-patient interaction and the individual level concerning health care, responsibility and actions. In the Swedish context, this focus conforms with guidelines of the National Board of Health and Welfare [65] website, which specify that “Good care is characterised by being person-centred, equal and safe”(our translation), as well as offering detailed recommendations concerning nurse-patient interaction.

As an explanation of individual emphasis more generally, Woolsey and Narruhn ([44] p. 607) suggest that that “It may be that advocating and addressing the needs of an individual patient feels more manageable [in education] than creating change at the systems level”. Neff et al. [45] also problematize whether we miss out helping students to understand health-related factors more holistically, since many decisions concerning health are not downsized to an individual level but rather need to be considered in a larger context including matters concerned with health care access, food sources, and housing, intertwined with policy and politics and economic systems. Overemphasis on the individual level will tend to obscure a societal and global perspective when learning about GH. Good health, as stated in Agenda 2030 [9], takes into account widening economic and social inequalities, as well as threats to the climate and the environment. Solely or mainly focusing on person-centred and individualised health care, without contextualising and positioning health and care in relation to society, nationally and globally, increases the risk for a perspective distortion and impedes taking responsibility for changes and development.

### 4.2. Lack of Theoretical Perspective

The lack of a theoretical framework increases the risk for approaching the subject matter purely based on opinions, personal values and preferences, without linking experiences based on personal background to a wider societal or global context. Our concern is that being able to take actions should follow an interpretation based on something other than personal values and backgrounds (cf. Valderama-Wallace and Apesoa-Varano, [66,67]), also with the risk that GH content becomes ‘fluid’ and related to partial issues, while not being related to any social context, understanding and explanations. In our data, we found that GH, as expressed in the course syllabi, are not understood, discussed or interpreted underpinned by any explicit theoretical approach. Without critical reflection and a theoretical framework, understanding of issues becomes fragmented, and will, in a worst-case scenario, provide support for maintaining inequality.

Several authors have discussed inequality, the need for combining many perspectives, and the pivotal role of power in GH and SDH (e.g., Solar and Irwin [26,27], Labonté and Ruckert [4]). As a possible theoretical framing of GH in nursing education, we suggest world-systems theory (WST), and turn to Wallerstein [33,34], Goldfrank [68] and Martinez Vela [69]. From this perspective, the economic disparities that underpin health inequity are not fortuitous but derive from the way relationships between states are structured. How well any country approaches understanding and managing GH issues can consequently never be considered a single nation-state issue according to WST. Understanding GH in nursing education in terms of ‘inequity and equity’ becomes clearer when using Wallerstein’s view, asserting that the world system can be considered as a social reality depending on the interconnection of nations. These relationships in turn derive from several components over time, including political science, economics and sociology, as well as historical processes. WST can be reinforced by the dependency theory [70], in how different levels of inequality are understood both between and within countries.

Empirical research (cf. Stoneman [71]) indicates that economic liberalization reforms between countries increases economic growth, but also involves that, “(1) the short-term association between investment of MNCs [Multi-National Corporations] and income growth is positive, whereas (2) the long-term association between the cumulated investment and growth is negative, and that (3) MNCs go together generally with higher income inequality”, Bornschier [72] (p. 191]). This means that even though some short-term positive effects occur, the outcome is that long-term global inequality is maintained, through the dependency between low- and high-income states [73,74]. Tausch [75], (p. 79) reanalyses and discusses Bornschier’s empirical 1980 study 20 years later, concluding that “dependence indeed has a critical impact on the over-all long-term development trends of a nation and that it tends towards the polarization of social relations”. Additionally, Bourguignon [76] (p. 14), contends that “in the developing world, economic liberalization reforms did not just enable the Global South to converge with the Global North, but also created new elites within those countries”. The increasing inequality within nations (in large related to this economical dependency among countries) is thus relevant to approaches in nursing education in relation to GH and SDH, inasmuch as a conventional incremental development agenda cannot remedy the deeper structural causes of health inequity (see also Labonté and Ruckert [4]).

### 4.3. Sustainability and Environment Related Content in the Course Syllabi

None of the programmes had a mandatory course dedicated to sustainability, environmental determinants of health or environmental justice, and no such course was listed as a possible elective in the programme descriptions. Wording relating to these issues was mostly quite vague, and besides a few references to Agenda 2030, reading lists lacked almost any reference to literature in the field. Mentions of sustainability were mostly made in the course syllabi here grouped under the themes Public Health, International Health, Health Promotion and Health and Caring, with a focus on social and economic aspects. In the course syllabi grouped under Traces, mentions concerned either the psychosocial environment, pharmaceuticals or antimicrobial resistance. Environmental justice was not explicitly addressed in any of the analysed syllabi.

### 4.4. Obstacles to including GH, EJ and SDH in Nursing Curricula

While numerous earlier studies point to the importance of including GH and SDH in nursing curricula, in order to move forward, a clearer understanding of the obstacles and opportunities is needed. Among the obstacles are the background of nursing educators and their relative status within their institutions. In a study of US nursing educators’ experiences teaching social justice content on undergraduate nursing programmes (Valderama-Wallace and Apesoa-Varano [67]), educators who wished to stress the issues felt isolated at their workplace and the educators’ backgrounds were significant for how content was taught. Some studies have been made on race and ethnic background among nurse educators [77,78], but we were unable to find research on nurse educators’ prior education with respect to sustainability studies, environmental determinants of health, personal experience of living in communities exposed to environmental hazards or prior experience working with environmental justice. We were also unable to find Swedish studies or national evaluations concerning race and ethnic background of nurse educators, their competence with respect to environmental and sustainability issues, if these aspects are considered in recruitment, or the role nurse educator background (see Valderama-Wallace and Apesoa-Varano [64,66,67]) plays in teaching global health.

A further obstacle noted in a study on Canadian educators’ perspectives on challenges and opportunities for integrating global citizenship and global health in the undergraduate nursing curriculum, was the reduction in electives students could choose from other disciplines and faculties [79], particularly the social sciences. In our study, a few programmes allowed electives in the social sciences, but we did not find any mention of electives oriented towards sustainability or EJ. The lack of a theoretical foundation/framework concerning the intention of learning about GH, EJ and SDH content in nursing education can explain the lack of electives relating to these issues.

### 4.5. Opportunities for Introducing GH, EJ and SDH in Nursing Curricula

Among the opportunities to introduce GH perspectives in undergraduate programmes, earlier research on nursing programmes suggests that theoretical content allowing students to grasp the structural meaning of health inequities, experiential activities and service-learning have proved effective. Baverstock et al. [80] observe that SDH receive little attention in programmes for the health professions and conclude that curricula should support students in understanding how to influence policy. In a study on UK and US student nurses’ attitudes to poverty and social justice, Scheffer et al. [81] found that teaching content on health inequities and SDH improved attitudes and student nurses’ confidence that they could take action. In a study by Woolsey and Narruhn [44], (p. 602), structural competency was used “as a tool to broaden the view of nursing students beyond individual, behavioural, biological, and cultural frameworks to encompass the structural determinants of health”.

Mohammed et al. [82] offer other examples of how social justice is taught in certain US undergraduate nursing programmes, with the aim of critically and morally developing students, and transforming them into agents of social change. In a US context, Schroeder et al. [83] underline the value of service-learning and spending time in the communities to offer experiential learning opportunities and support teaching of SDH in undergraduate nursing programmes. Several effective approaches used in teaching are summarised by Thornton and Persaud [84], who also stress the benefits of service-learning in the US. Quinn, El Ghaziri and Knight [85], as well as LeClair, Watts and Zahner [86] point to benefits of community partnerships. Patrick, Kingsley and Capetola [87] and Grose and Richardson [88] stress similar strategies for teaching environmental issues, and also underline social and environmental justice, as well as systems thinking.

### 4.6. Implications for Undergraduate Nursing Curriculum Development

In order to avoid not only a reductive understanding of GH but also the maintenance of an inadequate non-relational understanding, a theoretical framework is called for in undergraduate nursing curricula. We argue that world-systems and dependency theory have the potential to support nursing students in identifying adequate actions in their profession. Regardless of the theoretical framework opted for in nursing programmes, curricular content that includes policy, politics, economics and environmental justice, is necessary for making sense of contemporary global inequalities concerning health. A structural competency approach [43,44] appears promising, as well as service learning and course content oriented towards community health work. Compared to other countries, Swedish nursing education and health systems are not oriented towards community health. Decentralised primary health care centres exist, but these provide individual or family-centred care, while health promotion is generally a one-way process of informing individuals or targeted groups about diet, exercise or other life-style choices. Options in a Swedish context might therefore include connecting study-abroad periods to community health projects or using distance learning to connect to with community representatives and nurses working in partnership with communities in other countries.

Sufficient diversity in recruitment of nurse educators and students is crucial, as well as ensuring educators’ expertise with respect to EJ and environmental impacts. Organising continuous training of nurse educators is also needed to improve capacity. In those cases where expertise is lacking, attention to the selection of textbooks, and including articles, links to GH projects and other relevant material in the reading lists would at least enable students to independently deepen their understanding. We also agree with authors who stress the interrelationships between environmental justice, GH and SDH. Nursing education must take into account the widening economic and social inequalities and threats to the climate [86] and the environment. Climate change is likely to lead to an increase in disasters [89,90], demanding preparedness and practical skills among nurses [24,91,92]. Additionally, in this respect, partnerships with communities play a significant role for rapid interventions, communication, and post-disaster recovery, as well as for preventive measures and strengthening community resilience. Importantly, when integrating Agenda 2030 aims in nursing curricula, a more systemic approach is called for, to understand complex causal mechanisms, and to prepare nursing students to participate in the necessary transformation of health systems, in dialogue with communities and service users.

In the Swedish context, besides curriculum development at the level of individual HEIs, action could also be taken at the level of national guidelines and qualification requirements [63]. The wording of the nursing qualification requirements at the national level does not explicitly mention the need to consider global health dimensions. Requirements do specify that nurses should have an understanding of causes for health and ill health and that they should be able to relate to scientific evidence and theories. The fact that the scientific framing and types of causality considered are not explicitly described in the national requirements leaves the interpretation to the HEIs. The wording of the course syllabi further suggests that the scientific theories and evidence used as a basis for the programmes do not give a central place to global health or social, societal and equity questions in health more generally (cf. Brassolotto, Raphael, and Baldeo [93]). Other Swedish national documents indirectly regulate nursing education and practice (e.g., Prop. 2017/18:249 [94] which emphasises equity and social justice in public health; Skr. 2016/17:10 [95] sets the overarching aim of gender equality, while Prop. 2019/20:188 [96] reaffirms Sweden’s commitment to implementing Agenda 2030). Nevertheless, the SDH, environmental justice and structural causes of health inequity do not seem to be sufficiently clearly expressed to provide guidance (cf. Valderama-Wallace [97]). Finally, considering that most nations, including Sweden, have committed to implementing Agenda 2030, this could be a further avenue to drive curriculum development for GH.

### 4.7. Strengths and Limitations

The strength of our study lies in the explorative inductive approach, where all available course syllabi of Swedish HEIs offering undergraduate nursing programmes were scanned, so that even syllabi with minimal content relating to GH, EJ and SDH were included for analysis. The explorative inductive approach also meant that themes were not predefined but remained close to wording found in the syllabi. Our data collection and analysis included all available written material, programme descriptions, syllabi and reading lists, but we made no observations about how the syllabi intentions were carried out in terms of teaching and learning activities in the classroom or placements. Additionally, nurse educators may have used literature in their teaching practice that was not mentioned in the course syllabi and required reading lists. However, all HEIs in Sweden providing undergraduate nursing education were included and were reviewed, while reference literature on the reading lists was read to minimize the risk for any over-/under-interpretation of course content related to GH. Including all study places in Sweden (24 in all) makes it possible, according to our judgement, to give a reasonable and credible description of the current situation, concerning the intentions and content related to GH.

An important methodological clarification is that we did not make use of world-systems theory or dependency theory from the outset. As our study adopts an inductive-explorative approach, the ambition of this study was not to analyse our data using a theoretical framework, to avoid shoehorning data into any theory. However, at a later stage in the process, the results showed that a theoretical framework would benefit subject matter concerned with the intention of learning about content about GH, which is why these theories were included when discussing our results.

## 5. Conclusions

Social, environmental and structural determinants on health have a marginal position within the education in nursing, according to our results, and we suggest that GH, environmental justice and SDH subject matter become more central and explicitly integrated in the undergraduate nurse curriculum. There is a considerable risk in excluding societal and global perspectives when learning about GH by focus on the individual to an unconscionable extent, rather than understanding structural changes and reforms relevant on a societal–global level. How we understand ‘health’ therefore needs to be further developed and globally framed, underpinned by a theoretical and ontological understanding.

## Figures and Tables

**Table 1:** Overview of course syllabi according to year and number of core elements.

Total Number of Syllabi with Core Elements	Year 1	Year 2	Year 3
All core elements (*n* = 2)	1	2	
3–4 core elements (*n* = 18)	3, 4, 5, 6	2, 6 (*n* = 2), 7, 8, 9, 10	4, 6, 10, 11, 12, 13, 14
1–2 core elements (*n* = 25)	6, 8, 9, 10 (*n* = 2)	4 (*n* = 3), 7, 9, 10, 14, 18	6, 8 (*n* = 2)
	13 (*n* = 2), 15, 16, 17		11, 14, 18 (*n* = 2)
No core elements (*n* = 22)	1, 11, 12, 18, 19, 20, 21	2, 11, 19, 20, 21, 22, 23	4, 6, 12, 13, 14, 16, 17, 20

**Table 2:** Syllabi grouped under the theme Public Health.

Number of Core Elements	Programmes	Illustrative Excerpts
All core elements	1, 2	evaluate and compare different conditions for equal health, both nationally and globally/how inequity in health and care between men and women and between different socioeconomic groups arise due to traditions, culture norms and health/preventive and health promoting work/factors such as life circumstances, structural conditions, environment and lifestyle affect population health nationally and internationally, as well as health inequity
1–4 core elements	3, 4	good equal care in a developed public health policy/determinants of health, development and distribution of health, based on an individual, group, societal and sustainability perspective/epidemiology and public health/public health problems with a focus on risk factors for major disease
No core elements	19	

**Table 3:** Syllabi grouped under the theme International Health.

Number of Core Elements	Programmes	Illustrative Excerpts
All core elements	none	
1–4 core elements	2, 6, 7, 10, 11	public health work considering the global goals of gender equality, equality and sustainable development/identify and evaluate different health systems locally and globally/apply health promoting measures with a family and person-centred approach/ summarise and evaluate social health patterns from a political, demographic and socioeconomic perspective/ critically examine and analyse central areas in global health, such as poverty, inequity, gender, ethics, human rights and injury and violence/instruction will be given on the country’s health and care system
No core elements	21, 22	

**Table 4:** Syllabi grouped under the theme Health Promotion.

Number of Core Elements	Programmes	Illustrative Excerpts
All core elements	none	
1–4 core elements	5, 12, 13, 14	describe and identify conditions in society that affect the health of children and adults/problematise people’s living conditions and risk of ill health/problematise health/ill health based on factors such as biology, age, social cultural and economic differences, ethnicity, sexuality and gender/describe aetiology, pathophysiology, symptom, diagnosis and treatment of common infections/identify risk factors for ill health and disease with a focus on common diseases
No core elements	11	

**Table 5:** Syllabi grouped under the theme Health and Caring.

Number of Core Elements	Programmes	Illustrative Excerpts
All core elements	none	
1–4 core elements	4, 6, 9, 14, 15	explain basic health policy steering measures/structural conditions for health; health and care organisation, economics, politics, communication/account for the role and responsibility of nurses with respect to preventive and health promoting work, with a special focus on person-centred and culturally adapted care/international collaboration concerning health promoting work/how your own and societal attitudes, values and stances affect conditions for health

**Table 6:** Syllabi grouped under the heading Traces.

Number of Core Elements	Programmes	Illustrative Excerpts
1–4 core elements	4, 6, 7, 8, 9, 10, 13, 14, 16, 17, 18	public health work at an individual, group and societal level/disaster medicine is studied from a local and global perspective/discuss care in relation to sexual rights, including sexual violence, from a global and sustainability perspective, with a norm conscious approach/the concepts human rights, gender, equality and culture, with relevance in meeting the patient
Any other wording relating to GH	1, 2, 5, 6, 11, 12, 13, 14, 16, 17, 18, 19, 20, 21, 23	reflect on your own as well general attitudes and values with respect to gender and cultural differences in care/how aspects of ethnicity, age, class and gender can affect feelings of health and ill health, and how this can affect the care relationship/human rights are discussed in relation to men’s violence against women/the right to care on equal conditions
No wording	22, 24	

**Table 7:** Individual focus and theoretical content in the syllabi.

Content	Programmes	Illustrative Excerpts
Individual focus	1, 3, 6, 7, 8, 9, 10, 11, 12, 13, 14, 15, 16, 17, 18, 19	apply the care process based on person-centred care in collaboration with patient, relatives and other professions/describe health and safety promoting work, considering individuals’ physical and mental health and ill health/prepare a health promoting or preventive intervention at individual level based on evidence-based knowledge/reflect on the concepts of discrimination, abuse, equality and vulnerability relating to individuals’ life situation and needs
Theoretical content	2, 3, 4, 6, 8, 9, 12, 14, 18	problematise and propose how the content of global nursing and a global nursing approach can be developed in theory and in clinical practice in sustainable ways/generally relate to theories and concepts in care, medicine and public health work considering the global goals for gender equality, equality and sustainable development, with a focus on e-health/describe concepts, theories and models in public health science, public health work and epidemiology

**Table 8:** Overview of sustainability and environmental content in the syllabi.

Content	Programmes	Illustrative Excerpts
Sustainability, Agenda 2030, SDGs, environment	2, 5, 8, 9, 12, 13, 14, 15, 16	**General:** sustainable development: vision, concepts, definitions and state of the world/problematise sustainable development in a health promotion perspective at individual, group and societal levels/relate national health targets to the global goals for sustainable development/health in relation to sustainable development in society **Impacts of environment/sustainability on health:** sustainable development and its significance for human health/development /sustainable development and environmental effects on health/describe the consequences of current climate change based on the concepts ecological, economic and social sustainability as part of sustainable/the significance of the physical and psychosocial environment for health and ill health/argue for how resources, obstacles and risks in the environment impact human health **Impacts of health care on environment/sustainability:** resistance issues in connection with use of antibiotics linked to sustainable development/reflect on the responsibly of health care systems in the work towards the global goals for sustainable development

**Table 9:** Overview of sustainability and environmental content in the syllabi, more specifically in connection with health equity and/or global health.

Content	Programmes	Illustrative Excerpts
Sustainability more specifically in connection with health equity and/or global health	1, 2, 3, 6, 7, 10, 13, 15, 17, 18	**General:** gender equality, gender, equality and sustainable development/reflect on care in relation to gender, equality and sustainable development/reflect on global health challenges and the right to health from a gender, gender equality and sustainability perspective **Impact of environment/sustainability on health:** basic public health with a focus on factors such as life circumstances, structural conditions, the effects of environment and lifestyle on population health, both nationally and internationally, as well as health inequality/reflect on links between disease transmission, globalisation, poverty, life circumstances, environmental destruction and lifestyle in different parts of the world, and obstacles and opportunities for sustainable development in the area/describe the most important health factors in global health and their relation to the global goals for sustainable development **Action for sustainability in health care:** health and sustainability are studied in relation to the global and national sustainability and public health goals, where culturally adapted care and equal treatment are at the core of nurses’ health promoting work/health promotion and preventive care are treated from a local, national and global public health and sustainability perspective/create conditions for increased accessibility and sustainability in a flow and cost efficient manner/public health work considering the global goals for gender equality, equality and sustainable development/discuss sustainable development based on situations relating to safe care from a global perspective/apply principles for global nursing and care in accordance with the UN sustainability goals

## Data Availability

Programme descriptions and course syllabi are publicly available on the websites of Swedish HEIs offering undergraduate nursing programmes.
